# High density deposits of binary colloids

**DOI:** 10.1038/s41598-022-26151-9

**Published:** 2022-12-24

**Authors:** Hyoeun Kim, Marta Gonçalves, Sung Hoon Kang, Byung Mook Weon

**Affiliations:** 1 Soft Matter Physics Laboratory, School of Advanced Materials Science and Engineering, SKKU Advanced Institute of Nanotechnology (SAINT), Sungkyunkwan University, Suwon, 16419 South Korea; 2 Research Center for Advanced Materials Technology, Sungkyunkwan University, Suwon, 16419 South Korea; 3 Department of Mechanical Engineering and Hopkins Extreme Materials Institute, Johns Hopkins University, Baltimore, MD 21218 USA

**Keywords:** Colloids, Wetting

## Abstract

Colloids are essential materials for modern inkjet printing and coating technology. For printing and coating, it is desirable to have a high density of colloids with uniformity. Binary colloids, which consist of different size colloidal particles, have the potential to achieve high coating density and uniformity from size effects. We report a strategy to attain high-density deposits of binary colloids with uniform, crack-free, and symmetric deposits through droplet evaporation on micropillar arrays. We modify surfaces of micropillar arrays with plasma treatment to control their surface energy and investigate how binary colloidal fluids turn into well-controlled deposits during evaporation with X-ray microscopic and tomographic characterizations. We attribute temporary surface energy modification of micropillar arrays to the well-controlled high-density final deposits. This simple, low-cost, and scalable strategy would provide a viable way to get high-quality, high-density deposits of colloids for various applications.

## Introduction

Colloidal suspensions that consist of insoluble colloidal particles in a solvent are the main component of inkjet printing, a remarkably economical technique used for device manufacturing in several industries (i.e. optoelectronics, energy storage, tissue engineering, biodetection/sensing)^1–6^. Besides the economic advantage of inkjet printing, additional benefits come from the easy control of inkjet materials, delivering exact quantities of nanoinks through single ink droplets on pre-fabricated substrate patterns, and rendering specific structures and properties^7^. From a physical viewpoint, colloidal suspensions, including insoluble colloidal particles with diameters of approximately 1 nm to several $\upmu$m are considered a model of nanoinks^8,9^. The drying process of colloidal nanoinks is critical in achieving uniform deposition of colloidal particles but challenging in industrial applications because of coffee-ring effect, characterized by solute segregation at the contact line of evaporating colloidal droplets^10^, and crack formation, which is induced by air invasion into the deposits^11,12^. Both results cause nonuniform and defective deposition of colloids and eventually degrade the electrical and optical properties of deposits. Additionally, the evaporation of monodispersed colloidal nanoinks inevitably causes the generation of empty spaces among spherical colloidal particles^13^. The maximum packing density from monodispersed spherical particles is 74% when particles are arranged with face-centered or hexagonal closed packing, a crystalline state. The maximum packing density of a monodisperse particles with random packing is 64%^14^. In binary colloidal particles with two different diameters, small particles are expected to fill in empty space among large particles, increasing the packing fraction and generating a more physically stable deposition^15,16^. The increment of packing fraction by using bimodal particles, which leads to highly-dense deposits in the inkjet printing technique, has been shown to enhance electrical and sensing properties of printed devices effectively^17–19^. Finding appropriate deposition condition of binary colloids is crucial to improve coating density and uniformity of colloidal nanoinks deposits.

**Figure 1:**
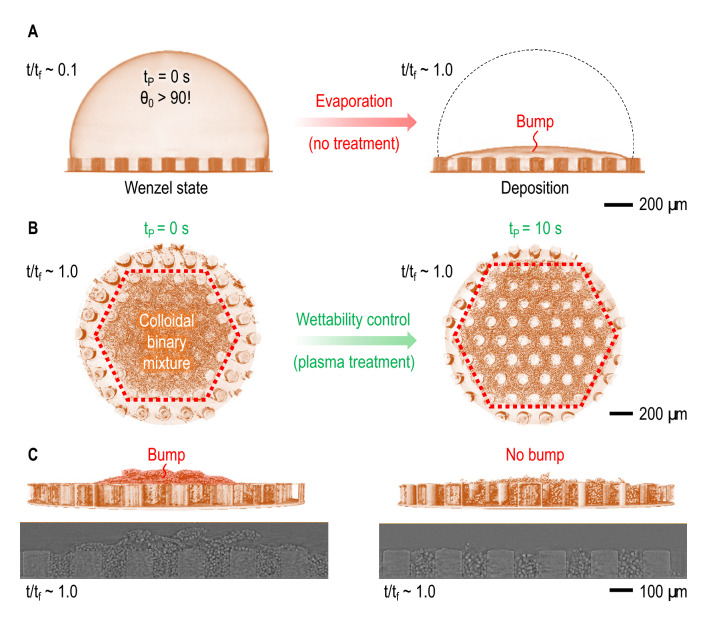
X-ray microtomography of evaporation-mediated deposition of binary colloids. (**A**) Starting colloidal droplets on PDMS micropillar patterns (left: at initial time $t/t_f \sim$ 0.1 ($t_f$ = evaporation complete time) with no treatment (initial contact angle $\theta _0 > 90^{\circ }$, showing Wenzel wetting state) results in bump formation after evaporation (right: at final time $t/t_f \sim$ 1.0). (**B**) By adopting plasma treatment for wettability control of micropillar patterns (plasma treatment time: $t_p =$ 10 s), symmetrically hexagonal-shaped uniform deposits are achieved after evaporation ($t/t_f \sim$ 1.0). (**C**) Bump formation after evaporation ($t/t_f \sim$ 1.0) is successfully prevented by adopting wettability control of micropillar patterns, as proven by the side-view (upper) and the cross-sectional (lower) images. All images generated with Amira software (version 2019.3).

Achieving uniform deposition of monodispersed colloids through evaporation is essential in recent studies^20,21^, including a micropillar-guided deposition^9^. Due to surface roughness, hydrophobicity of substrates^22–26^ would induce the self-pinning of colloidal fluids on micropillar substrates. A constant contact angle mode (CCA) during droplet evaporation would cause preferential accumulation of colloidal particles at the droplet center and eventually bump formation, as demonstrated in Fig. 1A. The existence of the bump at the droplet center results in the structural nonuniformity and instability responsible for the destruction and dysfunction of deposits. To prevent the bump formation from monodispersed colloids, we suggest a surface-modified micropillar-guided deposition for binary colloids to achieve uniform crack-free high-density deposition. As the surface energy controls droplet dynamics, controlling surface energy can provide opportunities to control deposition. The surface energy of the specimen is increased with plasma treatment, which increases the OH-terminal bonds^27,28^ and can induce the depinning of colloidal droplets on micropillar substrates, as demonstrated in Fig. 1B. The increasing surface energy spreads the droplet to pass through the pillar barriers, by which colloids can flow outward to the contact line. Eventually, the bump formation is successfully prevented by enhancing the surface wettability of micropillar substrates, as demonstrated in Fig. 1C.

This study demonstrates how to achieve symmetrically uniform crack-free hexagonal dense deposits of binary colloids using a surface-modified micropillar-guided deposition. Using high-resolution X-ray microtomography, we measure real-time dynamics of evaporation and deposition processes for binary colloidal droplets on plasma-modified micropillar substrates. Our findings contributes to identify the optimal conditions that are feasible in inkjet printing technology.

## Materials and methods

We examined two kinds of PDMS (polydimethylsiloxane) substrates for comparison: PDMS with flat surfaces and PDMS with micropillar patterns. The PDMS substrates were fabricated with SYLGARD$^{\textrm{TM}}$ 184 Silicone Elastomer Kit (Dow, USA) at a 10:1 ratio of polymer base to cross-linking curing agent. The PDMS substrates with micropillar patterns were fabricated using a silicon wafer master mold etched with the negative structure for cylindrical pillars arranged in a hexagonal pattern. The final geometry of the micropillar array was fixed in three dimensions: pillar diameter ($D_P$) = 100 $\upmu$m; pillar height ($H_P$) = 100 $\upmu$m; pillar pitch ($P_P$) = 100 $\upmu$m (see Fig. S1 of Supplemental Material). The pillar dimensions were chosen for optimal wetting modes, Wenzel modes, while ensuring polygonal depositions^9,29^. For the wettability control, the surface energy was increased by applying oxygen plasma treatment^27^. The plasma surface activation process was carried out with a Basic Plasma Cleaner (PDC-32G-2, Harrick, USA) at a radio frequency (RF) power setting of low (7.2W). The plasma treatment time ($t_p$) was increased from 5 to 25 s to determine the optimum surface energy.

An aqueous binary colloidal suspension was prepared by mixing $5.0 \pm 0.1$ vol$\%$ polystyrene particles of 2 $\upmu$m (small particles) and 10 $\upmu$m (large particles) (Polysciences, USA), showing a size ratio ($\omega _s$) to be 5 (large/small). Colloidal suspensions of polystyrene particles are widely used as model systems for inkjet printing studies^9,30,31^. Their surface chemistry is well controlled to be completely immersed and uniformly dispersed in solvents by the supplier. The basic knowledge taken from model particles would be useful in inkjet printing, despite differences in surface chemistry and ink formulation details. Overcoming a limit of monodispersed colloids, binary colloids with significantly different diameters would contribute to dense packing (Figs. S2, S3 of Supplemental Material). Tiny droplets with the initial volumes of $1.5 \pm 0.2$ $\upmu$L were carefully delivered with a micropipette on the PDMS substrates with different surface energies. The side-view optical images for contact angle measurements were acquired using a drop shape analyzer (DSA25, Krüss, Germany). A digital upright microscope (VHX-700FE, Keyence, Japan) was used to visualize the droplet’s temporal evolution and the final deposited pattern. Scanning electron microscopy (SEM) (S-3000H, Hitachi, Japan) was used to image the final binary colloidal deposits on the pillar substrate.

For in-situ observation of the evaporation process and visualization of the final deposited pattern of binary colloidal drops on micropillar patterned PDMS, high-resolution and high-speed X-ray imaging technique was employed in this study, allowing the acquisition of three-dimensional (3D) images through microtomography. The experiments were carried out at the 6C Bio Medical Imaging (BMI) beamline established in the Pohang Light Source (PLS-II), where the beam is composed of monochromatic synchrotron X-rays with 20 keV energy. A $\times$ 10 magnification was used, translating into an effective pixel size of 0.65 $\upmu$m and a field of view of 1.70 $\times$ 1.40 mm$^{2}$. For image acquisition, the sample is penetrated by the beam, followed by X-ray conversion into visible light by the scintillator (LuAG:Ce 50 $\upmu$m) and then received by the Scientific Complementary Metal–Oxide–Semiconductor (sCMOS) camera (pco.edge) (see Fig. S4 of Supplementary Material). For 3D image acquisition, 2D projections were acquired from 0 to 180 degrees while the sample was rotating, comprising a total of 900 projections. The Octopus Reconstruction software (Octopus, Belgium) was utilized to originate 2D tomographic slices from the X-ray projection images. The Octopus software also enabled image processing by applying noise and ring filtering. The 2D slices were processed with the visualization Amira Software (Thermo Fisher Scientific, USA) to achieve the three-dimensional volume images presented in this work. The Amira software was used for noise reduction and smoothing by applying a Median Filter to the 2D tomographic slices, the Volume Rendering function for the generation of the 3D images, and the dimensions of the pillars and colloidal deposits for analysis with built-in measurement tools^32^.

## Results and discussion

We successfully achieve highly uniform and dense deposits of binary colloids on surface-modified micropillar substrates, as described below.

### Droplet pinning dynamics on a plasma-modified substrate

**Figure 2:**
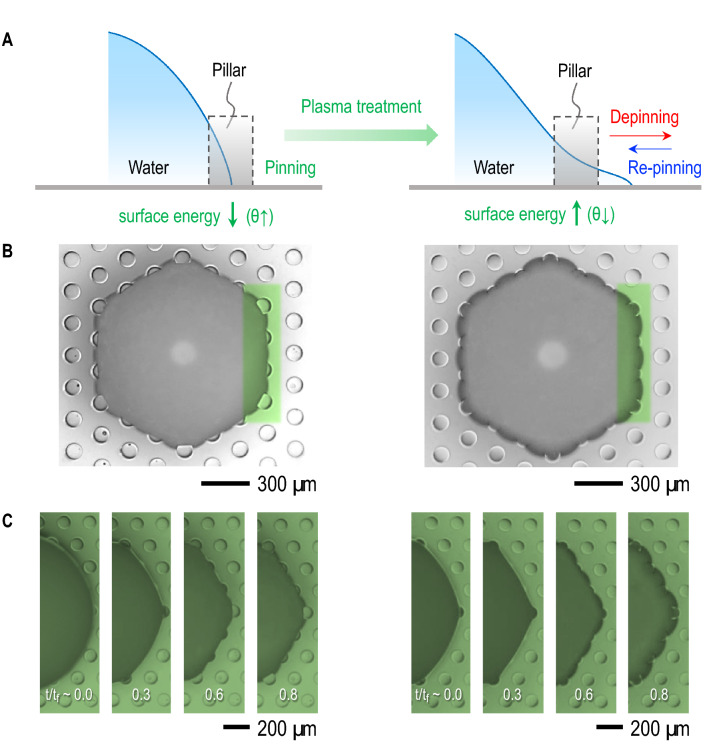
Pinning, depinning, and re-pinning dynamics of samples with and without surface modification through plasma treatment. (**A**) Initial low surface energy (or large contact angle) substrates is favorable for pinning of colloidal fluids between pillars (left) and plasma treatment to increase surface energy or decrease contact angle of substrates is essential for depinning to pass through between pillars (right). The final deposit shape is determined by re-pinning of the contact line. (**B**) Optical microscopic images for no treatment (left) and plasma treatment for 10 s (right) (both images were taken at $t/t_f \sim$ 0.7) demonstrate contributions of pinning (left) and depinning (right) between pillars (Acquired with VHX-700FE, Keyence). (**C**) Temporal evolution of pinning by no treatment (left) and of depinning and re-pinning by plasma treatment for 10 s (right).

The initial depinning dynamics, as illustrated in Fig. 2A, is explained by surface energy modification through plasma treatment contribution, which is essential to control the initial pinning and depinning dynamics of binary colloidal fluids. Initial low surface energy or large contact angle of micropillar substrate with no treatment would be favorable for initial pinning of colloidal fluids between pillars, as demonstrated in Fig. 2B (left). Plasma treatment increases surface energy (or decrease contact angle), which is essential to induce initial depinning that makes contact lines pass through pillars, as shown in Fig. 2B (right). The temporal evolutions of pinning with no treatment and depinning with plasma treatment are shown in Fig. 2C. Surface energy modification by plasma treatment is critical for pinning or depinning dynamics at initial times.

**Figure 3:**
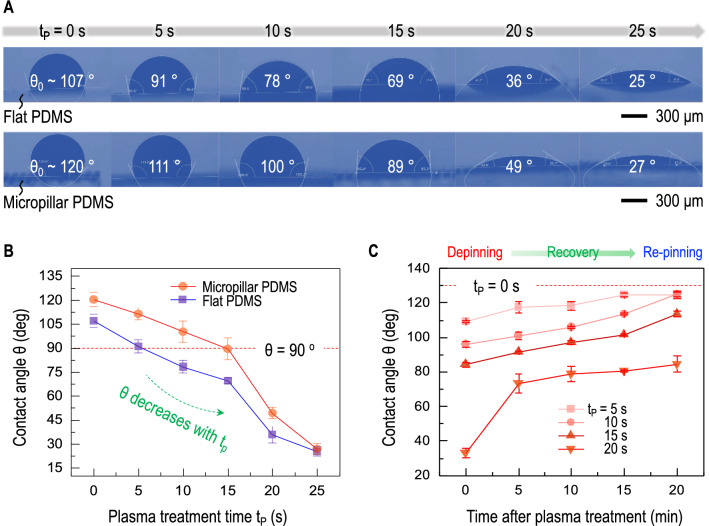
Wettability control with plasma treatment. (**A**) Initial contact angles decrease with increasing plasma treatment time ($t_p$) for both flat and micropillar PDMS (Acquired with Drop Shape Analyzer and Advance Software, Krüss Science). (**B**) Initial hydrophobicity ($\theta > 90^{\circ }$) turns into hydrophilicity ($\theta < 90^{\circ }$) at $t_p \sim 5$ s for flat PDMS and at $t_p \sim 15$ s for PDMS micropillars. (**C**) Hydrophilicity transition by plasma treatment is temporary, and recovery to hydrophobicity is shown in contact angle changes with time after plasma treatment. Early hydrophobicity is favorable for depinning, contributing to flattening the upper bump, and late recovery is favorable for re-pinning, contributing to the final deposit shape by surface area minimization.

The late re-pinning dynamics, as illustrated in Fig. 2A, is attributed to that hydrophilicity or less hydrophobicity by plasma treatment is temporary and recoverable to original hydrophobicity. Contact angle modification with plasma treatment time ($t_p$), as summarized in Fig. 3A, demonstrates that initial contact angles decrease by plasma treatment times for both flat and micropillar PDMS substrates. The gradual decrease of contact angles by plasma treatment times turns hydrophobicity ($\theta > 90^{\circ }$) into hydrophilicity ($\theta < 90^{\circ }$), as demonstrated in Fig. 3B. The transition from hydrophobicity to hydrophilicity appears at $t_p \sim 5$ s for flat PDMS and at $t_p \sim 15$ s for micropillar PDMS. Most importantly, hydrophilicity or less hydrophobicity with plasma treatment is not permanent. Hydrophilicity is therefore recovered to hydrophobicity, as observed in Fig. 3C. The recovery of oxygen plasma treatment is well known as aging effect^33–37^. Original hydrophilicity is favorable for initial depinning, contributing to flattening the upper bump. Late recovery from hydrophilicity to hydrophobicity is favorable for re-pinning, minimizing the final deposit surface area.

The recovery dynamics of oxygen plasma treatment by aging effects is due to air exposure under ambient conditions ($22 \pm 3\,^\circ$C room temperature and $35 \pm 5$% relative humidity). The longer plasma treatment time leads to a longer recovery time. In particular, micropillar PDMS substrates for $t_p = 5$ s and $t_p = 10$ s is fully recovered to $\theta _0 \sim 120^\circ$, 20 min after plasma treatment. During the evaporation process, the droplet coverage prevents the local hydrophilicity transition on the liquid–solid interface by avoiding the substrate contact with air^34^. However, the air exposure allows the surrounding substrate to be prone to induce the aging effect, facilitating the recovery dynamics. The recovery dynamics of micropillar substrates are responsible for the late re-pinning dynamics and thus the final minimum deposit surface area.

### Achieving highly-dense uniform deposits from binary colloids

The high-density packing of binary colloids requires the uniformity of the deposition. An appropriate mixture of binary colloids is expected to realize a high-density coating if small particles sufficiently fill the space between large particles^16^. The size ratio ($\omega _s$) and the mixing volume ratio ($\omega _m$) between small and large particles are essential to obtain the highest packing density. For a colloidal mixture that displays binary particle dynamics, it is necessary to have a size ratio larger than 1.4^15^. We find low-density deposition by monodispersed-like behaviors at small size ratios and size separation (Figs. S3, S5 of Supplemental Material). Size separation for evaporating droplets, including binary colloids, can be induced due to hydrodynamics by diffusion force during evaporation^10^. We choose the size ratio to be $\omega _s = 5.0$ (= 5/1 = large/small) and the mixing volume ratio to be $\omega _m = 0.5$ (= large/total) to achieve dense, uniform and crack-free deposits.

**Figure 4:**
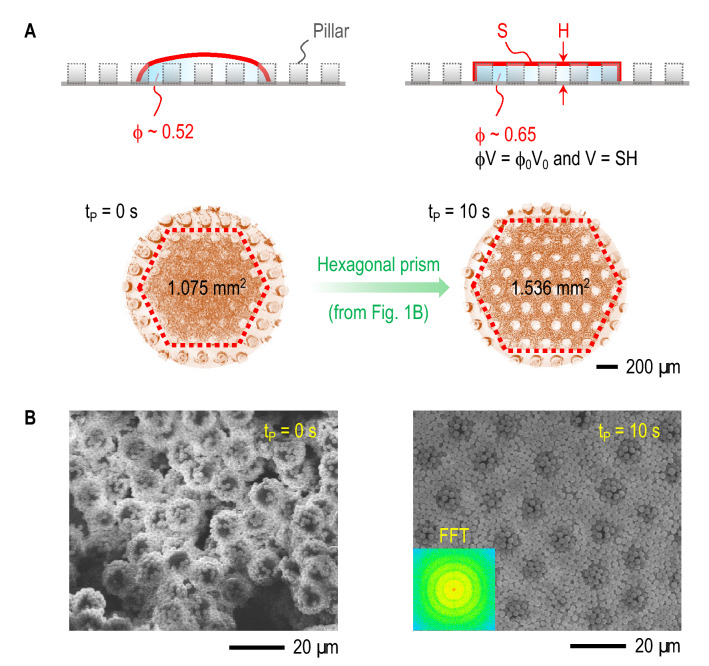
High-density deposition of binary colloids. (**A**) Schematic illustration with bump (left) and without bump (right): volume conservation ($\phi V=\phi _0 V_0$) and hexagonal prism model ($V = SH$) suggest high-density deposition of binary colloids with the final packing density $\phi \sim 0.65$ by the plasma treatment ($t_p = 10$ s) (Generated with Amira software (version 2019.3)). (**B**) Scanning electron microscopy (SEM) images on the deposit top layers: (left) large particles surrounded by small particles behave like single-sized large particles, and (right) small particles fill voids between large particles, creating dense packing (Acquired with S-3000H, Hitachi). The FFT (fast Fourier transform) of the right SEM image, taken by ImageJ, indicates that binary colloids build randomly packed noncrystal structures (inset). Optimal high-density packing is achieved when small and large particles are uniformly mixed by the mixing volume ratio of $\omega _m = 0.5$ (= large/total).

For the specified conditions and without plasma treatment ($t_p = 0$ s), the bump formation will be present, depicting a higher height (*H*) of the deposit due to the highly constricted surface area of the hexagonal prism base (*S*) (Fig. 4A, left). The bump formation, a failure of the uniform deposition, (Fig. 4B, left) induces the low particle density deposition of the bump layer, where large particles surrounded by small particles may behave like single-sized large particles, hindering a high-density deposition. We visualize the discontinuously arranged particles in Fig. 4B (left), which would result in poor electrical property, a weakness for inkjet-printed device fabrication^38^. Overcoming the bump emergence is possible with plasma treatment, leading to a final pattern with uniform height, distributed through a wider surface area, effectively removing the bump and exposing the pillar top surfaces evenly, in opposition to the absence of plasma treatment (Fig. 4A, right). Here, the size of small particles is sufficiently little to fill voids between the large particles (Fig. 4B, right). Eventually, the deposit pattern can stabilize without a bump and prevent crack formation (Fig. S6 of Supplemental Material).

Hexagonal deposit patterns with geometrical uniformity can be expected by the hexagonal prism model^9^, where the hexagonal deposit volume is obtained as $V = SH$ and assuming the volume conservation principle for the total particles volumes $\phi V=\phi _0 V_0$ (the final particle packing fraction $\phi$, the initial particle volume fraction $\phi _0$, and the initial droplet volume $V_0$). The two main parameters, *S* and *H*, are quantified in our experiments in (Fig. 4). *H* is taken on average from the side-view X-ray imaging, for instance, $H \sim$ 155 $\upmu$m with bump and the average $H \sim$ 100 $\upmu$m without bump (Fig. 1C). *S* is taken by subtracting the total surface areas of the pillar tops inside the hexagonal prism from the surface area of the hexagonal prism base, for instance, $S = 0.934$ mm$^{2}$ ($= 1.075{-}0.141$) with bump and $S = 1.159$ mm$^{2}$ ($= 1.536{-}0.377$) without bump. For both cases, $V_0 = 1.5$ mm$^{3}$ and $\phi _0 = 0.05$. The final packing fraction of the binary colloidal deposit for the two cases can be then expected by

1 $$\begin{aligned} \phi =\frac{\phi _0 V_0}{S H}, \end{aligned}$$

where the bump deposit pattern reveals a lower final packing fraction of $\phi = 0.52 \bigg ( =\frac{0.05 \times 1.5}{0.934 \times 0.154}\bigg )$ in comparison to the uniform deposit pattern, which accounts for a packing fraction of $\phi = 0.65 \bigg (=\frac{0.05 \times 1.5}{1.159 \times 0.100} \bigg)$. The estimated $\phi$ values reinforce the main strength of binary colloids, since $\phi$ is effectively increased for both cases, compared with the previous value for monodispersed deposits of $\phi = 0.48$^9^.

**Figure 5:**
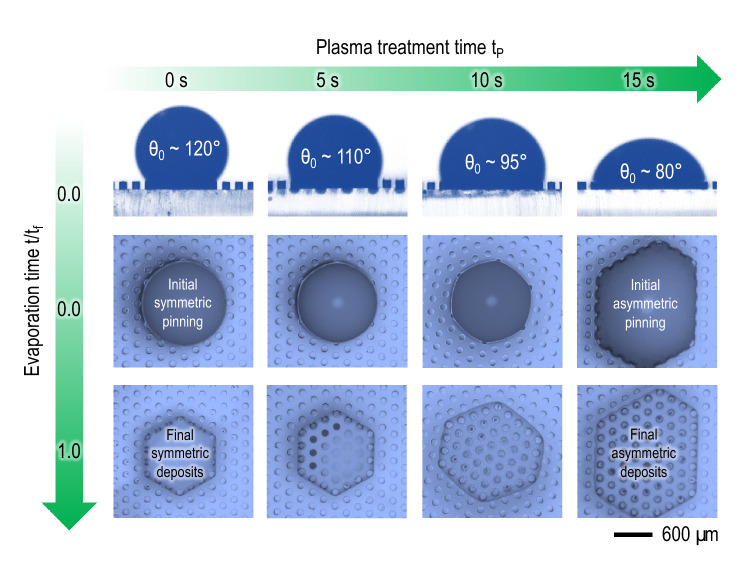
Symmetric deposition by initial symmetric pinning. Initial contact angles larger than 90$^{\circ }$ (upper row) are essential for initial symmetric pinning (middle row) and, therefore, final symmetric deposits (lower row) (Acquired with VHX-700FE, Keyence and Drop Shape Analyzer and Advance Software, Krüss Science).

The symmetric deposition is achieved by initial symmetric pinning, as illustrated in Fig. 5. Initial contact angles determined by plasma treatment times $t_p$ can be larger than 90$^{\circ }$, causing initial symmetric pinning by the capillarity predominance. This initial symmetry results in the final bilateral symmetric deposits. We often observe bump formation for $t_p = 0$ or 5 s and asymmetric deposits for $t_p = 15$ s. Consequently, initial hydrophobicity and temporal hydrophilicity transition for $t_p = 10$ s are critical to finally achieving symmetrically uniform crack-free hexagonal deposits. The complete evaporation dynamics affected by the substrate wettability attained for each $t_p$ is represented in Fig. S7 of Supplemental Material. The attainable symmetric patterns enhance local uniformity of particles distribution, ensuring a steady packing fraction in any direction. Furthermore, this well-controlled evolution of the polygonal contact line is crucial for optimized textured surfaces in printing applications^39^.

### Optimized conditions for high-quality deposit patterns

The optimum mixtures in binary colloids are crucial to achieve highly-dense uniform patterns. The colloidal suspension, being a binary mixture of particles, has two main criteria: particle size ratio ($\omega _s$ = large particle radius divided by small particle radius) and binary mixing volume ratio ($\omega _m$ = large particle total volume divided by total particle volume). Regarding the optimum $\omega _s$ values, to avoid the monodisperse-like behavior of larger particles, the size ratio should be $\omega _s > 1.4$ in our experiments. Simultaneously, for larger size ratios ($\omega _s > 6.46$)^15^, gravity can induce percolation of small particles, leading size separation (Fig. S3 of Supplemental Material). Previous experimental and theoretical studies suggest that a size ratio of $\omega _s \approx 5$ contributes to a system with more uniform distribution and low mobility allowing increased jamming, corroborating our option^14,16^. Concerning the mixing volume ratio $\omega _m$, a volume fraction of large particles to the total volume of dried binary particles, if smaller particles are included in a greater ratio, patterns are more prone to crack due to air invasion at the final evaporation stage (Fig. S5 of Supplemental Material)^8,40^. Furthermore, a mixture with $\omega _m \approx 0.7$ would form a packed structure, consistent with previous works^15,16^. However, the micropillar array hydrophobicity allied to the presence of larger particles would lead to an enhanced movement of smaller particles outwards to the drying interface, constraining the larger particles to stack at the center^10,16,41^. The size separation at $\omega _m = 0.75$ can be a setback leading to the bump formation (Fig. S5 of Supplemental Material). Therefore, the approach of $\omega _m = 0.5$ is justified by the final goal of uniformity of the deposit.

Finally, we discuss packing fraction differences before and after plasma treatment. Our results indicate that the higher packing fraction is present for the uniform deposit pattern, consistent with the observation in Fig. 4B (right), where the pillars play the role of imprisonment leading to a cohesive pattern. In contrast, the lower packing fraction present in the bump pattern is mainly due to the size separation, displaying a non-desirable monodisperse-like behavior of larger particles (Fig. 4B, left), since smaller particles preferentially move outwards^10,16^. Theoretically, a reachable final packing density is known as $\phi = 0.71$ for randomly packed binary colloids^15^, while the high-density deposits are achieved as $\phi = 0.65$ in this work. This discrepancy is due to hydrodynamics effects in evaporating droplets, where the flows disturb uniform particle distribution, which can induce colloids to be randomly packed rather than crystallized. We achieved a low packing density of 0.48 from randomly packed monodispersed colloids^9^. Therefore, binary colloids are more favorable for a high packing density than monodispersed colloids. Our finding on the deposition dynamics will be relevant to the evaporation dynamics of binary colloids, for instance, regarding evaporation dynamics on superhydrophobic surfaces^42^. Further studies are required to understand evaporation dynamics and to overcome hydrodynamic effects during evaporation to increase deposit densities.

## Conclusion

Our study represents a feasible method to achieve high-quality, high-density hexagonal deposits of binary colloids on hexagonal-patterned micropillar substrates. Temporarily turning the substrates hydrophilic with oxygen plasma treatment helps to induce the initial depinning dynamics. The late recovery to hydrophobicity is helpful to induce the re-pinning dynamics of binary colloidal fluids on the substrates. These controlled dynamics would be crucial to achieve symmetric high-density deposits of binary colloids. Additionally, the proposed method forms horizontally uniform heights by removing height differences between the center and the edge of final deposition patterns when using binary colloids and micropillar patterns. The achievable increased packing fraction imply numerous advantages for industrial performances^43–45^. This strategy would help to optimize inkjet printing by proceeding with inexpensive, easy-fabricated, well-controlled high-density deposits of binary colloids for many possible applications, being nearly universal.

## Supplementary Information

Supplementary Information.

## Data Availability

The data that supports the findings of this study are available within the article and its Supplementary Material.
