# Supplementary material for: High density deposits of binary colloids
Source: Sci Rep. 2022 Dec 24;12:22307. doi: 10.1038/s41598-022-26151-9 (PMC9790000; doi:10.1038/s41598-022-26151-9)
Supplement: Supplementary file 1 — Supplementary Information. [file 41598_2022_26151_MOESM1_ESM.pdf]

High density deposits of binary colloids: Supplementary Information

Hyoeun Kim[1,2,3,+], Marta Gonçalves[1,+], Sung Hoon Kang[3,*], and Byung Mook Weon[1,2,*]

[1]Soft Matter Physics Laboratory, School of Advanced Materials Science and Engineering, SKKU Advanced Institute of Nanotechnology (SAINT), Sungkyunkwan University, Suwon 16419, South Korea
[2]Research Center for Advanced Materials Technology, Sungkyunkwan University, Suwon 16419, South Korea
[3]Department of Mechanical Engineering and Hopkins Extreme Materials Institute, Johns Hopkins University, Baltimore, MD 21218, USA
[*]email: shkang@jhu.edu
[*]email: bmweon@skku.edu
[+]these authors contributed equally to this work

ABSTRACT

Colloids are essential materials for modern inkjet printing and coating technology. For printing and coating, it is desirable to have a high density of colloids with uniformity. Binary colloids, which consist of different size colloidal particles, have the potential to achieve high coating density and uniformity from size effects. We report a strategy to attain high-density deposits of binary colloids with uniform, crack-free, and symmetric deposits through droplet evaporation on micropillar arrays. We modify surfaces of micropillar arrays with plasma treatment to control their surface energy and investigate how binary colloidal fluids turn into well-controlled deposits during evaporation with X-ray microscopic and tomographic characterizations. We attribute temporary surface energy modification of micropillar arrays to the well-controlled high-density final deposits. This simple, low-cost, and scalable strategy would provide a viable way to get high-quality, high-density deposits of colloids for various applications.

S1. Micropillar array design

Figure S1 shows the micropillar array organization and their dimensions through scanning electron microscopy [Fig. S1(a)] and X-ray imaging [Fig. S1(b)]. There are three different dimension parameters that can be defined: pillar diameter (D_P), pillar height (H_P) and the closest distance between pillars called pitch (P_P).

S2. Area fraction difference with monodisperse and bidisperse particles

Figure S2 shows the scanning electron microscope images (S-3000H, Hitachi, Japan) of monodispersed [Fig. S2(a), left] and bidispersed [Fig. S2(a), right] colloidal deposit patterns and respective analysis with the Fiji program (Fiji, GNU General Public License). The obtained information makes it possible to know the number of particles and the area occupied by the particles. For monodispersed large colloids (10 μm), the total particle area was 6171 ± 1 μm^2, while for binary colloids, the total particle area was 7543 ± 1 μm^2. By dividing the occupied area by the total area of the image (8015 ± 1 μm^2), the area fraction can be calculated. Our approach to binary colloids implies an area packing density increment from 0.77 to 0.94. This estimation suggests that binary colloids are more favorable for dense packing than monodispersed colloids.

S3. Binary colloidal deposits in function of particle size ratio

Figure S3 shows the X-ray microtomography images of different particle size ratio (ω_s) deposits. Large particle size was fixed as 10μm and smaller particle size changed from $\omega_s = 10.0$ (= 10:1 = large/small) to 5.0 (= 5:1) and 3.3 (= 3.3:1).

S4. X-ray microscopy imaging

Figure S4 shows a schematic illustration of the image acquisition process with X-ray computed microtomography. The complete explanation of the process can be found in the Materials and Methods section.

S5. Binary colloidal deposits in function of particle mixing volume ratio and initial concentration

Figure S5 shows the X-ray computed microtomography images of different particle mixing volume ratios (ω_m) [Fig. S5(a)] and initial concentration [Fig. S5(b)]. As the amount of small particles increases, deposit pattern destruction from crack-formation due to air invasion at the final evaporation stage is dominant. In the case of initial concentration, if the initial concentration is too low, it causes void areas within the deposit pattern, which is a negative phenomenon to achieving a uniform pattern. However, if it is too high, colloidal particles are highly stacked and eventually form thick deposition, which is sensitive to crack formation.

S6. Uniformity comparison before and after plasma treatment

Figure S6 shows the scanning electron microscopy images before [Fig. S6(a)] and after [Fig. S6(b)] plasma treatment. If the colloidal droplet evaporates on the superhydrophobic substrate, a bump appears in the center of the final deposit pattern, which leads to eventual destruction because of a structurally unstable state.

S7. Wettability-dependent evaporation dynamics

Figure S7 shows the side-view time-lapse data acquired for evaporating droplets with a Drop Shape Analyzer. The distinct states of wettability were achieved with different plasma treatment times (t_p), leading to the contrasting evaporation dynamics of droplets on the micropillar substrates.

(a)

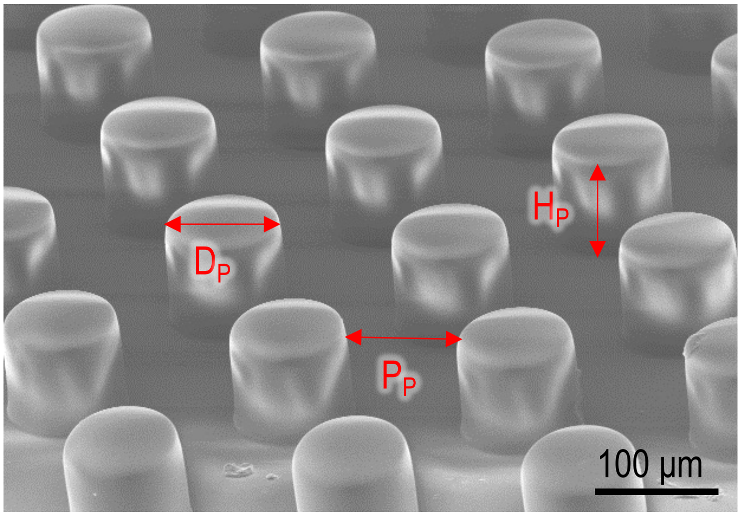

(b)

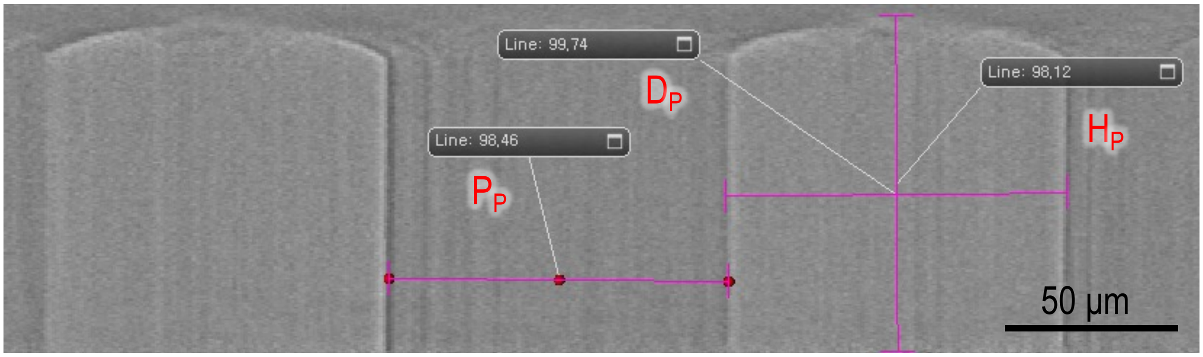

Figure 1. PDMS micropillar patterned substrate observation and dimensions with (a) SEM imaging and (b) X-ray computed microtomography.

(a)

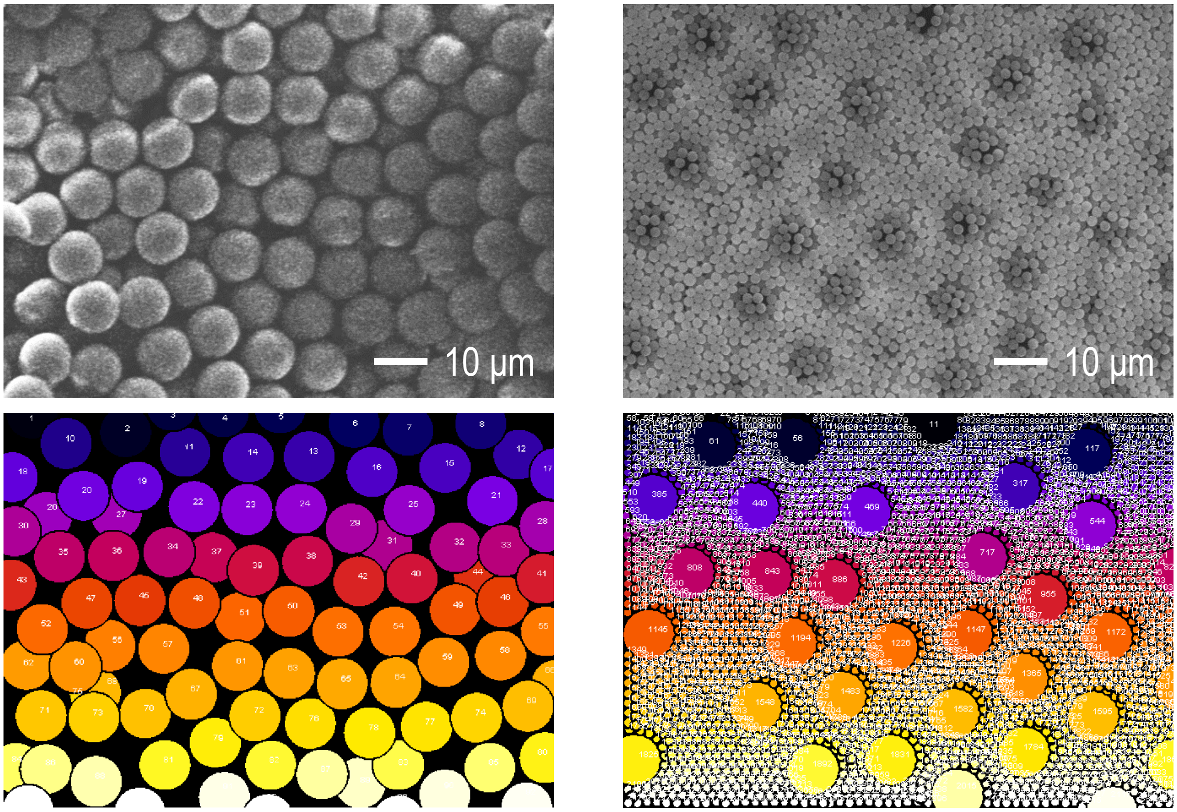

(b)

	Monodispersed	Binary
Number of particles	96	2217
Particle area (μm^2)	6171.72	7543.22
Total area (μm^2)	8015.90	8015.90
Area fraction	0.77	0.94

Figure 2. SEM image for area packing density comparison between (a) monodisperse (10 μm) (left) and bidisperse (10 μm + 2 μm, 1:1 ratio) (right) with (b) calculated values. Initial concentration: 5.0 ± 0.1 vol%; Droplet initial volume: 1.5 ± 0.2 μL.

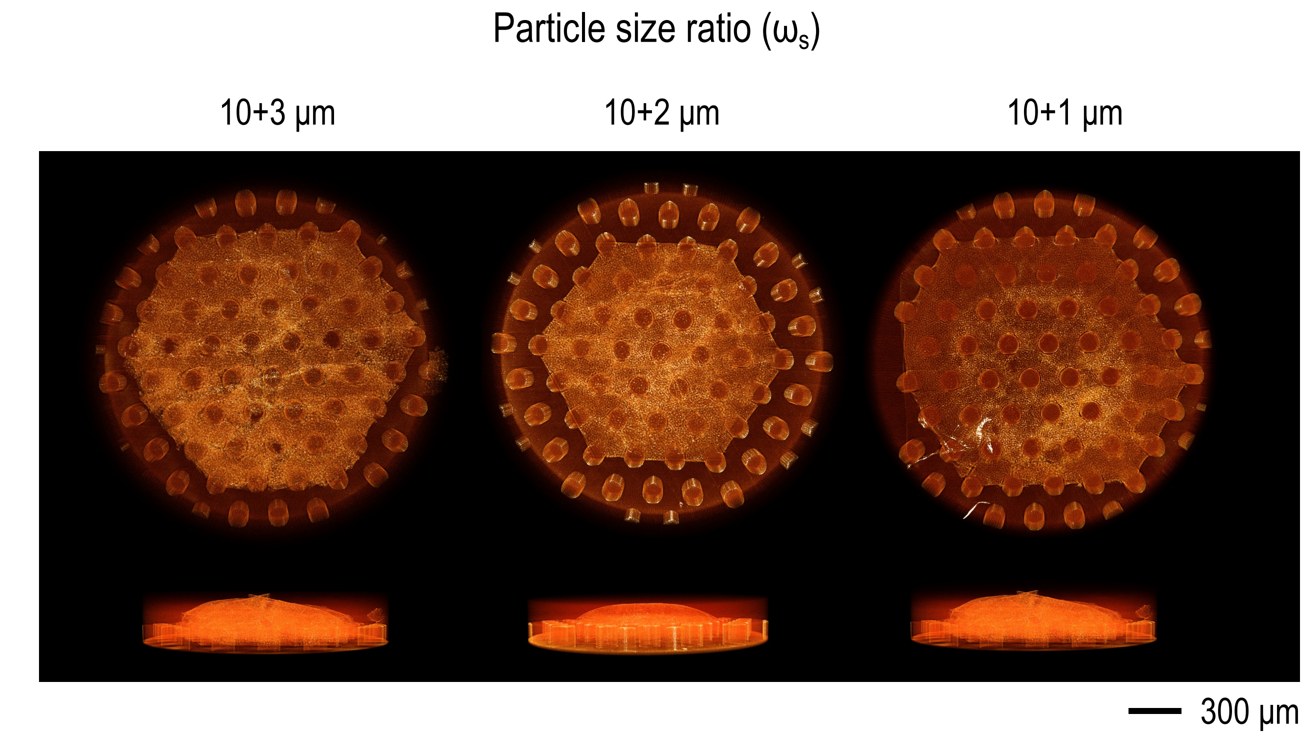

Figure 3. X-ray computed microtomography image of colloidal deposit with particle size ratio difference. Initial concentration: 5.0 ± 0.1 vol%; Particle mixing ratio: 1:1 (larger : smaller); Droplet initial volume: 1.5 ± 0.2 μL.

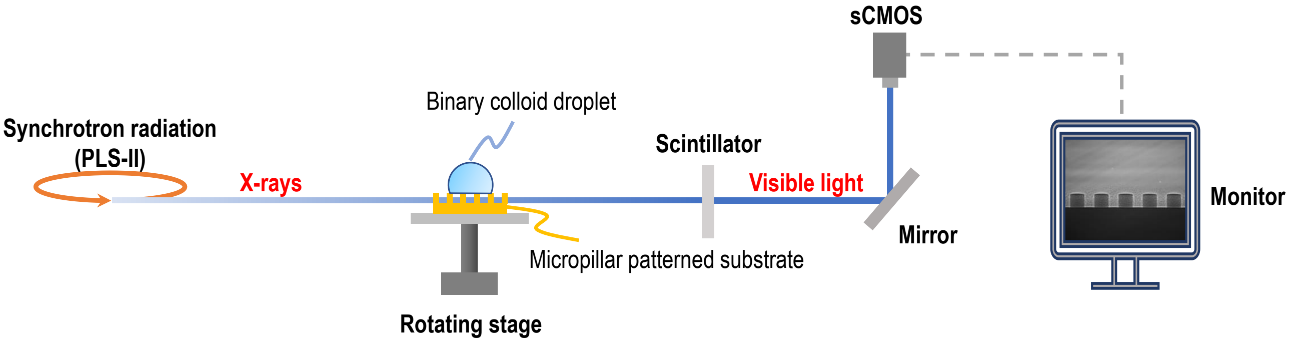

Figure 4. Schematic illustration of X-ray microscopy technique.

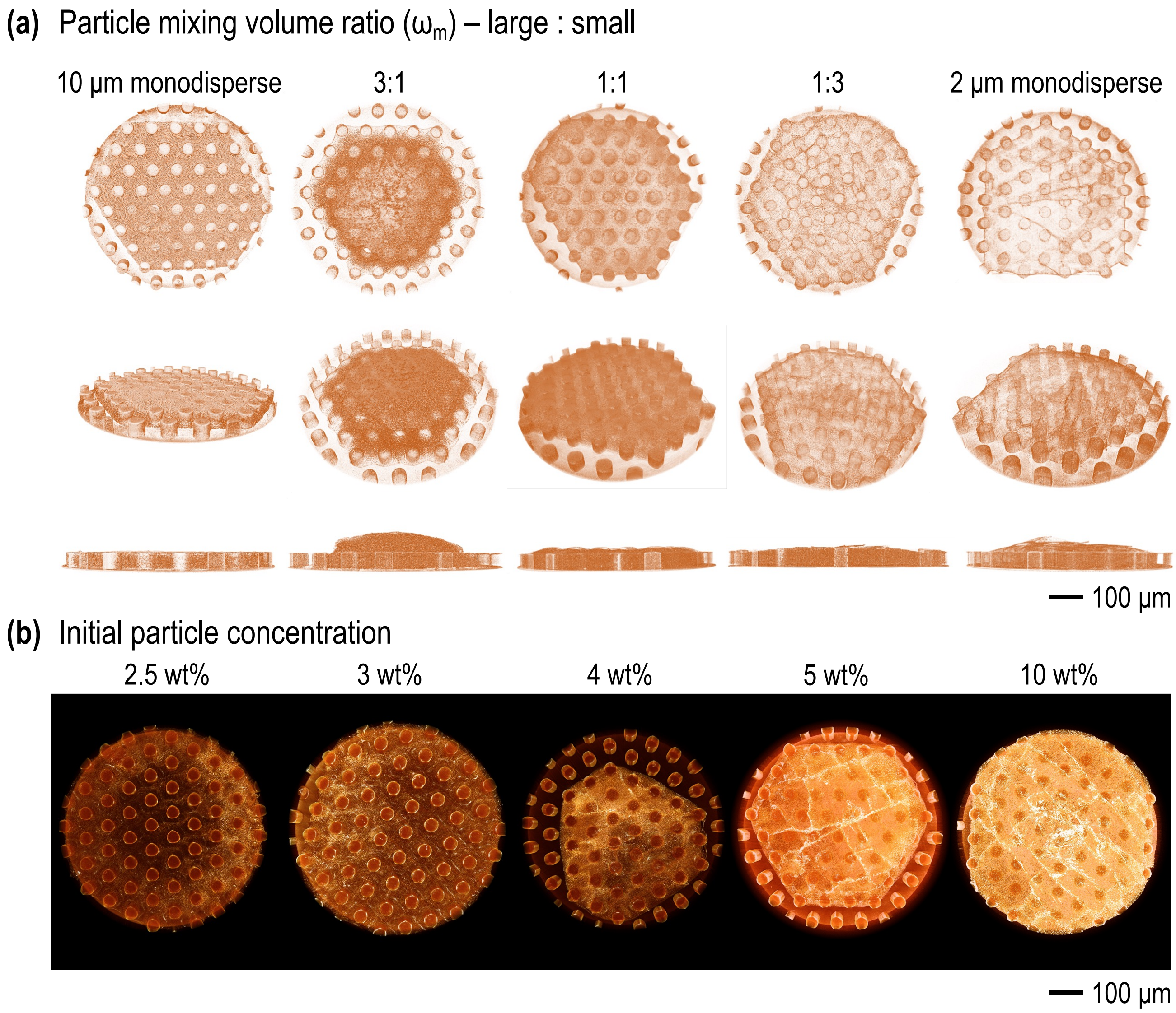

Figure 5. X-ray microtomography image of the colloidal deposit depends on (a) particle mixing ratio and (b) initial concentration. Droplet initial volume: $1.5 \pm 0.2\ \mu$L.

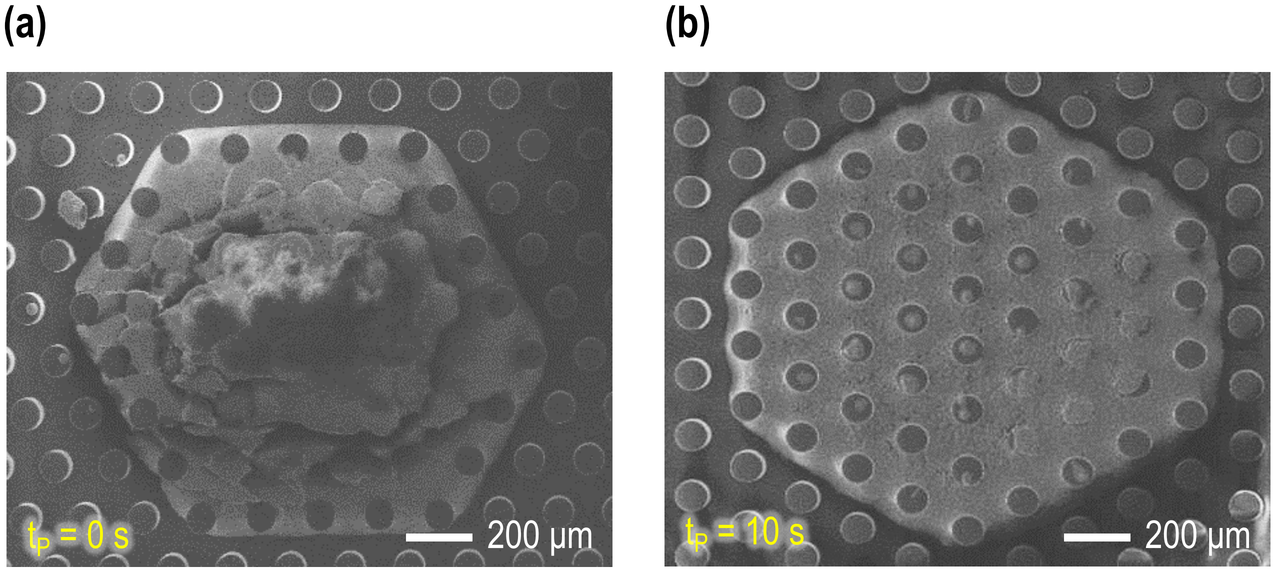

Figure 6. SEM image of colloidal deposit pattern (a) with bump and (b) without a bump. Initial concentration: 5.0 ± 0.1 vol%; Particle mixing ratio: 1:1 (10 μm + 2 μm); Droplet initial volume: 1.5 ± 0.2 μL.

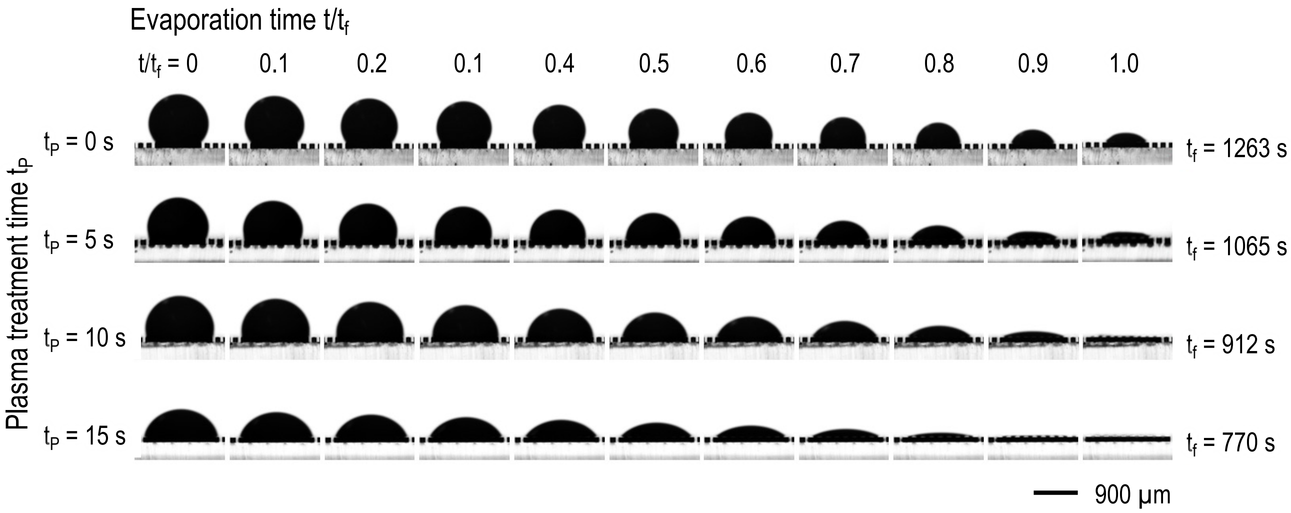

Figure 7. Side-view visualization of wettability-dependent evaporation dynamics of droplets on micropillar substrates subjected to different plasma treatment times (t_p).
